# Different characteristics of cardiac biomarkers to decide and predict the culprit lesions in patients with suspicious acute coronary syndrome

**DOI:** 10.1007/s00380-015-0698-5

**Published:** 2015-06-17

**Authors:** Mitsunobu Kitamura, Noritake Hata, Tadateru Takayama, Atsushi Hirayama, Masashi Ogawa, Akira Yamashina, Hisaaki Mera, Hideaki Yoshino, Fumitaka Nakamura, Yoshihiko Seino

**Affiliations:** Division of Intensive Care Unit, Nippon Medical School Chiba Hokusoh Hospital, 1715, Kamagari, Inzai, Chiba 270-1694 Japan; Division of Cardiology, Department of Medicine, Nihon University School of Medicine, Tokyo, Japan; Department of Cardiology, Tokyo Medical University, Tokyo, Japan; Second Department of Internal Medicine, Kyorin University School of Medicine, Tokyo, Japan; Third Department of Internal Medicine, Teikyo University Chiba Medical Center, Chiba, Japan; Cardiovascular Center, Nippon Medical School Chiba Hokusoh Hospital, Chiba, Japan

**Keywords:** High-sensitivity troponin T, Heart-type fatty acid-binding protein, Acute coronary syndrome, Coronary angiography, Culprit lesion

## Abstract

This multicenter prospective study was conducted to assess high-sensitivity troponin T (hs-TnT) and other biomarkers to decide and predict culprit lesions indicated for emergency percutaneous coronary intervention (PCI) in patients with suspicious acute coronary syndrome (ACS). We have reported Hs-TnT is the most sensitive biomarker for earlier diagnosis and decision making in patients with suspected ACS. In this study, we had conducted subanalysis investigating the usefulness for prediction of ACS culprit lesion. The patients with suspicious ACS and initially negative whole-blood rapid troponin T test, who underwent coronary angiogram (CAG), were enrolled (*n* = 74). Hs-TnT, quantitative assay for conventional troponin T (c-TnT), creatine kinase MB isozyme (CK-MB), and heart-type fatty acid-binding protein (H-FABP) were simultaneously measured. ACS culprit lesion was described as total occlusion, subtotal occlusion, and/or angiographical unstable lesion such as thrombosis, ulceration or irregularity. The CAG revealed that 49 cases had ACS lesions to be indicated for emergency PCI. The areas under the ROC curves and ROC-optimized cut-off of hs-TnT, c-TnT, CK-MB, and H-FABP were 0.75, 0.67, 0.68, and 0.75, respectively, and 18, 11, 2.0, and 4.6 ng/ml, respectively. In patients with total occlusion and 90–99 % of diameter stenosis (TIMI 2 or 3), hs-TnT could predict emergency PCI with significantly higher sensitivity compared with H-FABP (hs-TnT >14 ng/ml; 71 %, and H-FABP >6.2 ng/dl; 51 %, *p* = 0.021) and other biomarkers. Meanwhile, H-FABP displayed significant correlations with number of diseased vessels and presence of thrombotic lesion. The present study first revealed different characteristics of correlation between the angiographic culprit lesions and each cardiac biomarker. For prediction of ACS lesions requiring emergency PCI, hs-TnT had the highest sensitivity with satisfied analytical precision.

## Introduction

An early diagnosis is of absolute necessity for appropriate therapeutic decision and risk stratification in patients with acute coronary syndrome (ACS). Cardiac troponin measurement has become essential in the diagnosis of acute myocardial infarction (AMI) according to the ESC/ACC Task Force redefinition of myocardial infarction [1]. Moreover, as the Joint ESC/ACC/AHA/WHF Task Force presented in the universal definition of myocardial infarction in 2007 [2], the diagnosis of AMI is accurately determined based on a rise and/or fall in cardiac biomarkers with at least one value above the 99th percentile of the upper reference limit.

We have reported the excellent diagnostic performance of high-sensitivity troponin T (hs-TnT) in comparison with other cardiac biomarkers in the HsTnT-iNET (High-Sensitivity cardiac Troponin T for earlier diagnosis of acute myocardial infarction in patients with an Initially NEgative Troponin T test) study [3]. In brief, the HsTnT-iNET study demonstrated that hs-TnT displayed the highest sensitivity among the validated cardiac markers, that is the sensitivity and NPV of hs-TnT were both 100 % for AMI diagnosis after 120 min from the onset.

The coronary angiography (CAG) as well as cardiac biomarker has been a gold standard for the contemporary practice to diagnose and to make the therapeutic decision for ACS. However, there was little evidence in the investigation of cardiac biomarkers in comparison with the findings of CAG. Therefore, we conducted an additional analysis to investigate the diagnostic performance of the cardiac biomarkers to predict presence of culprit lesions for emergency percutaneous coronary intervention (PCI) in ACS.

## Materials and methods

### Study design and population

The present HsTnT-iNET substudy was a prospective multicenter study including 5 participating tertiary centers equipped with coronary care units in Japan. From November 2009 through January 2011, patients who presented at the emergency room with symptoms suggestive of AMI and initially negative TnT were enrolled in the present study. This study was conducted according to the principles of the Declaration of Helsinki and the protocol was approved by the ethics committee at each participating institution. Written informed consent was obtained from all patients in the emergency room.

Final diagnosis was adjudicated based on the initial diagnosis and diagnosis at discharge from the hospital, and electrocardiogram (ECG) changes during hospitalization. In the present CAG study, we conducted an additional subanalysis, and the raw data of ECG and CAG were collected to analyze precise correlations between the cardiac biomarkers elevation and the culprit lesions characteristics.

**Fig. 1:**
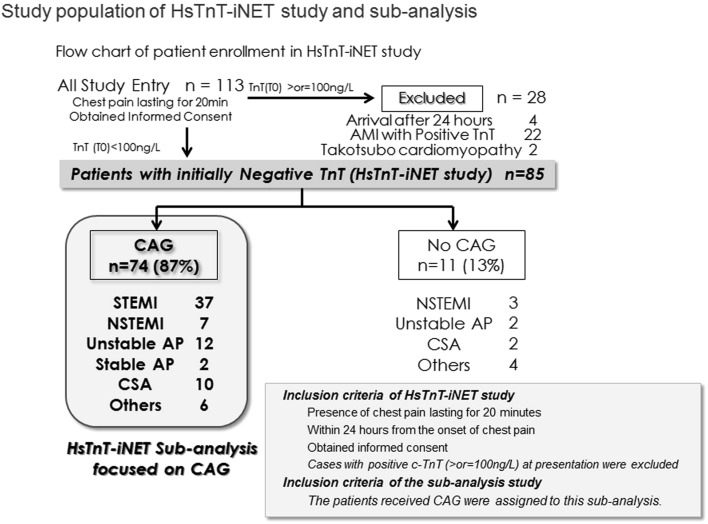
Study population of HsTnT-iNET study and subanalysis

Out of 85 patients with negative c-TnT test (<100 ng/L) assigned to the HsTnT-iNET study, total of 74 patients who underwent CAG were included in this CAG analysis (Fig. 1). The baseline characteristics of the patients and the details of diagnosis are presented in Table 1. The median age was 70 years (IQR 66–75). The median time from the onset of symptoms to drawing of first blood sample was 135 min (IQR 99–165). Ischemic ST-T findings of ECG were confirmed in 62 patients (84 %) at presentation, with ST-segment elevation being most frequently detected [*n* = 44 (59 %)]. The details of treatments within 48 h from presentation are shown in Table 1. Thirty-seven patients (50 %) were classified as having ST-segment elevation MI (STEMI) and 7 patients (9 %) as having non-ST-segment elevation MI (NSTEMI). There were 40 patients (54 %) in Killip class I, 3 (4 %) in class II, and 1 (1 %) in class III. Type 4a MI was not detected in the present study population.

**Table 1:** Baseline characteristics of study population

Age, year		Pharmacological therapy within 48 h, *n* (%)		
Median	70	Antiplatelet		70 (96)
Interquartile range	66–75	Anticoagulant		65 (89)
Male sex, *n* (%)	67 (91)	Thrombolytic agent		0 (0)
Risk factors, *n* (%)		Nitrates		27 (37)
Hypertension	61 (82)	Beta blocker		20 (27)
Hyperlipidemia	41 (55)	RAA blocker		33 (45)
Diabetes	18 (24)	Ca Channel Blocker		15 (21)
Current smoking	27 (36)	IV-Nicorandil		36 (49)
Past history, *n* (%)		IV-Inotropes		8 (11)
Coronary artery disease	18 (24)	Mechanical support, *n* (%)		
Previous MI	4 (5)	Pacemaker		12 (16)
Previous PCI	8 (11)	IABP		5 (7)
Previous CABG	1 (1)	ECLS		0 (0)
Status of arrival, *n* (%)		Invasive treatment, *n* (%)		
Walk-in	15 (20)	Coronary angiography		74 (100)
EMS	55 (74)	PCI		52 (70)
Helicopter EMS	5 (7)	CABG		0 (0)
Referral from other clinic	15 (20)	Final diagnosis, *n* (%)		
Time from onset of chest pain, min		AMI		44 (59)
Median	165	STEMI		37 (50)
Interquartile range	100–327	NSTEMI		7 (9)
Electrocardiographic findings at presentation, *n* (%)		Killip	Class I	40 (54)
ST-segment elevation	44 (59)		Class II	3 (4)
ST-segment depression	11 (15)		Class III	1 (1)
T-wave inversion	7 (9)		Class IV	0 (0)
Abnormal Q wave	10 (14)	Non-AMI		30 (41)
No significant findings	12 (16)	Unstable AP		12 (16)
eGFR		Stable AP		2 (3)
Median, ml/min	76	VSA		10 (14)
Interquartile range, ml/min	60–88	Other cardiac		1 (1)
eGFR < 60 ml/min, *n* (%)	18 (24)	Non-cardiac		3 (4)
eGFR < 30 ml/min, *n* (%)	2 (3)	Unknown		2 (3)

*MI* myocardial infarction, *EMS* emergency medical service, *eGFR* estimated glomerular filtration rate, *RAA* renin–angiotensin–aldosterone, *IABP* intra-aortic balloon pumping, *ECLS* extracorporeal life support, *PCI* percutaneous coronary intervention, *CABG* coronary artery bypass graft, *AP* angina pectoris, *VSA* vasospastic angina

### Blood sampling and validated cardiac biomarkers

Blood samples were drawn during the acute phase, at presentation (T0), 3, and 6 h after the T0 sampling. If the whole-blood rapid TnT test was positive (conventional TnT≧100 ng/L) at presentation (T0), the patients were excluded from the present study. Immediately after centrifugation, the sample serum was stored at −80° until each measurement was performed. The levels of conventional troponin T (c-TnT), and creatine kinase MB isozyme (CK-MB) were measured by an electrochemiluminescence immunoassay with the Roche Modular Analytics E170^®^. Hs-TnT was measured by an electrochemiluminescence immunoassay with the ECLusys^®^/Elecsys^®^ hs-TnT 2010 Roche diagnostics assay. The analytical characteristics of the troponin assays have been reported at http://www.ifcc.org. In the c-TnT assay, the 99th percentile of the upper reference limit, coefficient of variance at the 99th percentile, and the value with a 10 % coefficient of variance were calculated to be 10 ng/L, 18 %, and 30 ng/L, respectively. Therefore, the cut-off value for a diagnosis of AMI was defined as 30 ng/L because the precision was unsatisfactory at the 99th percentile. In the CK-MB assay, the 97.5th percentile of the upper reference limit was reported to be 3.1 ng/ml, and the coefficient of variance at 8.2 ng/ml was calculated to be 3.1 %. The cut-off value of CK-MB at 5.0 ng/ml had been used for the diagnosis of AMI, which fulfilled required analytical precision. The serum concentration of heart-type fatty acid-binding protein (H-FABP) was measured by an enzyme-linked immunosorbent assay.

The cut-off values of hs-TnT, c-TnT, CK-MB, and H-FABP were defined as 14 ng/L (the value at 99th percentile upper reference limit), 30 ng/L (the value for 10 % coefficient of variance), 5.0, and 6.2 ng/L. The values of CK-MB and H-FABP had been currently used as the cut-off point to diagnose MI in the clinical practice.

### Coronary angiography

All patients enrolled in this subanalysis underwent CAG within 48 h from the onset of symptom. A significant stenosis was defined as a percentage of diameter stenosis (%DS) > 75 %, and a severe stenotic lesion with 99–100 % stenosis was classified by Thrombolysis in Myocardial Infarction (TIMI) flow grade. (TIMI flow grade 3; full perfusion with normal flow, grade 2; perfusion of the entire vessel with delayed flow compared with a normal artery, grade 1; some penetration of the contrast material beyond the point of obstruction but without perfusion of the distal coronary bed, grade 0; complete occlusion) [4] In this study, the antegrade flow with TIMI flow grade 0 or 1 was defined as total occlusion, and that with TIMI flow grade 2 or 3 was defined as subtotal occlusion. The collateral filling developed for the severe stenosis with total or subtotal occlusion was described as the grades reported by Rentrop (Collateral filling grade 0; none, grade 1; filling of side branches of the artery via collateral channels without visualization of the epicardial segment, grade 2; partial filling of the epicardial segment via collateral channels, grade 3; complete filling of the epicardial segment of the artery via collateral channels) [5].

Angiographic ACS lesion was defined as total occlusion, subtotal occlusion, or moderate stenosis with thrombosis, ulceration or irregularity. When the culprit lesion was not located in the main coronary vessel (right coronary artery, left main trunk artery, left anterior descending artery or left circumflex artery), but in the branch perfusing the lateral myocardium (i.e., diagonal branch, obscure marginal branch, intermediate artery), the culprit vessel was described as lateral branch. The CAG findings were interpreted by 2 interventional cardiologists (MK, NH) without the background information regarding cardiac biomarkers in the core laboratory.

### Adjudication of the final diagnosis

To determine the final diagnosis for each patient, all case records collected from all participant institutes were reviewed, regarding the clinical diagnosis, coronary risk factors, time course from the onset of symptoms to presentation, findings of the ECG at presentation and 24 h later, coronary angiography (CAG), and other examinations and all medical treatments. The diagnosis of AMI was determined according to the universal definition of MI as we recently reported [3]. Vasospastic angina (VSA) was diagnosed for the patients with resting chest pain in the situations of no significant organic stenosis explaining AMI or presence of the CAG-proved coronary spasm (>75 %) with or without provocation test (acetylcholine or ergonovine) [6–8]. We basically adopted the final diagnosis declared from the participating institution, if there was no discrepancy between the diagnosis and the collected clinical findings. In patients with non-ST-segment elevation MI (NSTEMI) who received percutaneous coronary intervention (PCI) before the second blood sampling, the cut-off point of c-TnT was defined as three times the 99th percentile URL (90 ng/L), because myocardial injury could not be discriminated between AMI and peri-procedural necrosis [9].

### Interventional procedures

Interventional procedures for reperfusion were selected at operator’s discretion, included thrombectomy, balloon dilatation, stent implantation, and distal protection. We reviewed use of thrombus aspiration, stent, drug-eluting stent for revascularization, and final TIMI flow grade after the procedure. Temporary pacing, intra-aortic balloon pumping, and extracorporeal life support were indicated if necessary.

### Statistical analysis

Continuous variables are presented as the means (±standard deviation) or medians [the interquartile range]. Categorical variables are presented as numbers and percentages. The diagnostic performances of each assay were represented as the sensitivity, specificity, positive predictive value (PPV), negative predictive value (NPV), and receiver operating characteristics (ROC) curve with the area under the curve, presented together with the 95 % confidential interval (95 % CI) or ±standard error. Comparisons between biomarkers were performed by an analysis of the differences of the diagnostic performances with McNemar’s test, Fisher’s exact test or Pearson’s Chi-square test, as appropriate. The statistical analysis of differences between the area under the ROC curve of each biomarker was performed using the method reported by Hanley et al. [10]. Kruskal–Wallis test was used for an overall analysis among more than 3 groups, then if statistically significant, Mann–Whitney *U* test was performed to compare each other. These analyses of differences between biomarkers were adjusted by Bonferroni correction within the multiple comparisons for the flow status of the culprit lesion, the culprit coronary artery group, and the number of diseased vessels. The statistical analyses were performed using the SPSS^®^ software program version 20.0.0 (IBM^®^ Corporation, New York, NY, USA). The values of the validated cardiac biomarkers were described as box plot in figures to describe the differences of the various CAG findings. Values of *p* < 0.05 were considered to indicate statistical significance.

## Results

### Angiographic finding and final diagnosis

Angiographic findings of the study population are shown in Table 2. The CAG study revealed that 25 patients (34 %) had total occlusion as the culprit lesions. Those with total occlusion and poor collateral (Rentrop’s collateral flow grade 0 or 1) were all diagnosed as STEMI (*n* = 19). In those with total occlusion and good collateral (grade 2 or 3), there were 3 patients with STEMI, 2 with NSTEMI, and one with unstable AP. In the all patients with subtotal occlusion (*n* = 12), good collateral flow (grade 2 or 3) was not observed, and there were 9 patients with STEMI, and 3 with unstable AP. In those with severe stenosis of %DS 90–99 % but TIMI flow grade 3, there were 3 patients with STEMI, 4 with NSTEMI, and 5 with unstable AP. There were 2 patients with STEMI who showed CAG-proved spastic stenosis but no organic stenosis. In those with culprit stenosis of %DS 0–75 %, there were one patient with STEMI, one with NSTEMI, 3 with unstable AP, 2 with stable AP, 5 with VSA, and 6 without CAD. With the more severe lesion in angiographic finding, the more frequently the patient was diagnosed as AMI (Table 2).

**Table 2:** Final diagnosis and angiographical result of study population

All patients (*N* = 74)							
Diameter stenosis of the culprit lesion	All *n* = 74 (100)	100 % (TIMI 0–1)		99 % (TIMI 2)	90–99 % (TIMI 3)	Spasm	0–75 %
		Rentrop 0–1	Rentrop 2–3	Rentrop 0–1			
		*n* = 19 (26)	*n* = 6 (8)	*n* = 12 (16)	*n* = 12 (16)	*n* = 7 (9)	*n* = 18 (24)
Adjudicated diagnosis							
AMI	44 (59)	19 (100)	5 (83)	9 (75)	7 (58)	2 (29)	2 (11)
STEMI	37 (50)	19 (100)	3 (50)	9 (75)	3 (25)	2 (29)	1 (6)
NSTEMI	7 (9)	0	2 (33)	0	4 (33)	0	1 (6)
Unstable AP	12 (16)	0	1 (17)	3 (25)	5 (42)	0	3 (17)
Stable AP	2 (3)	0	0	0	0	0	2 (11)
VSA	10 (14)	0	0	0	0	5 (71)	5 (28)
Non CAD	6 (8)	0	0	0	0	0	6 (33)
The details of angiography							
Spasm provocation test	5 (7)	0	0	0	0	4 (57)	1 (6)
Diseased vessel							
RCA	25 (34)	10 (53)	4 (50)	6 (50)	2 (17)	2 (29)	1 (6)
LAD	29 (39)	7 (37)	1 (17)	3 (25)	5 (42)	7 (100)	6 (33)
LCX	8 (11)	1 (5)	1 (17)	2 (17)	3 (25)	1 (14)	0
LMT	0 (0)	0	0	0	0	0	0
Lateral branch	6 (8)	1 (5)	0	1 (8)	2 (17)	0	2 (11)
Number of diseased vessel							
No significant stenosis	10 (14)	0	0	0	0	0	10 (56)
1-vessel disease	50 (68)	14 (74)	4 (67)	12 (100)	10 (83)	5 (71)	7 (39)
2-vessels disease	13 (18)	4 (21)	2 (33)	0	2 (17)	1 (14)	1 (6)
3-vessels disease	2 (3)	1 (5)	0	0	0	1 (14)	0
Type of culprit lesion							
Thrombotic	34 (46)	19 (100)	5 (83)	8 (67)	2 (17)	0	0
Complex	13 (18)	0	1 (17)	5 (42)	5 (42)	0	2 (11)
Revascularization	52 (70)	19 (100)	6 (100)	12 (100)	11 (92)	0	3 (17)
Thrombus aspiration	29 (56)	19 (100)	5 (83)	5 (42)	0		1 (33)
Stent	46 (88)	18 (95)	6 (100)	11 (92)	11 (92)		2 (67)
Drug-eluting stent	8 (11)	1 (5)	1 (17)	2 (17)	3 (25)		1 (33)
CABG	0 (0)	0	0	0	0		
Final TIMI flow grade 3	46 (88)	16 (84)	6 (100)	9 (75)	11 (100)		3 (100)
Grade 2	6 (12)	3 (16)	0	3 (25)	0		0
Grade 0–1	0 (0)	0	0	0	0		0
Max CPK in AMI patients	*n* = 44	*n* = 19	*n* = 5	*n* = 9	*n* = 7	*n* = 2	*n* = 2
Median	2010	2658^†^	2911	2133	195^†^	414	655
IQR	676–2913	2010–2983	720–2917	962–2898	151–438	265–562	378–932

*AMI* acute myocardial infarction, *STEMI* ST-segment elevation myocardial infarction, *NSTEMI* non-ST-segment elevation myocardial infarction, *AP* angina pectoris, *VSA* vasospastic angina, *CAD* coronary artery disease, *RCA* right coronary artery, *LAD* left anterior descending artery, *LCX* left circumflex artery, *LMT* left main trunk artery, *CABG* coronary artery graft bypass

^†^ *p* value <0.05 in max CPK

### Cardiac biomarkers with stratification by angiographic findings

**Fig. 2:**
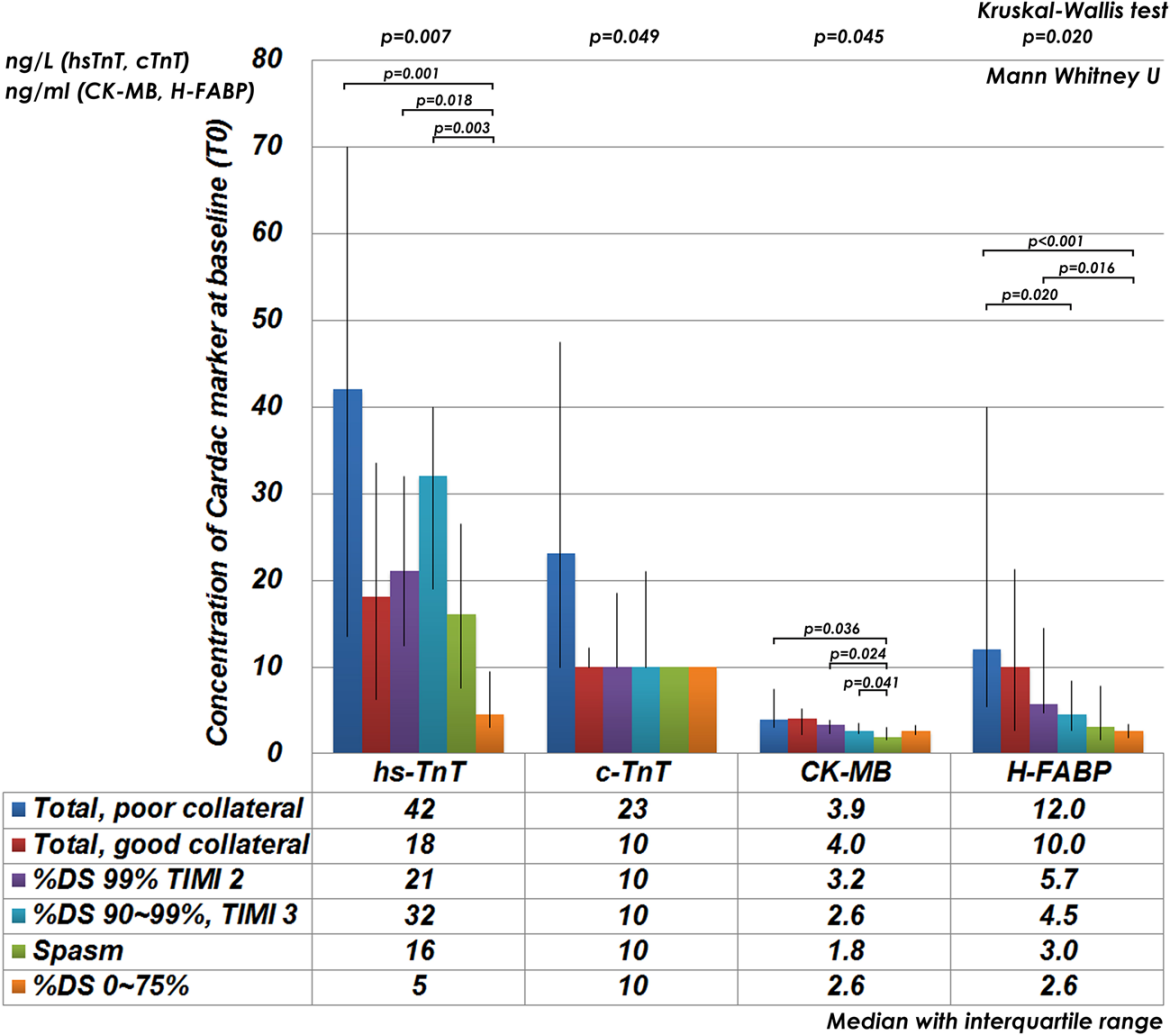
Cardiac biomarkers with stratification by severity of the culprit lesion

The concentrations of the cardiac biomarkers were stratified by angiographic severity of the culprit lesion (Fig. 2). Those with the total occlusion and poor collateral showed significantly higher values of hs-TnT compared with those in other culprit lesions; however, c-TnT did not discriminate difference in the other angiographic findings. H-FABP discriminated those with total occlusion regardless of collateral grade, but did not discriminate those with significant stenosis with subtotal occlusion or %DS 90–99 %. In those with spastic stenosis, only hs-TnT increased higher than the cut-off value (i.e., 14 ng/L).

In patients with severe obstructive lesions (total occlusion, subtotal occlusion (TIMI 2), and %DS 90–99 % TIMI 3), hs-TnT presented positive in 35 of 49 patients (71 %), meanwhile H-FABP presented positive in 25 of 49 patients (51 %) (*p* = 0.021). Moreover, in patients with %DS 90–99 % which were all diagnosed as AMI or UAP, hs-TnT presented positive in 11 of 12 patients (92 %); however, H-FABP presented positive in only 5 of 12 patients (42 %) (*p* = 0.063). Overall, hs-TnT could diagnose severe obstructive lesions with 72 % of accuracy with a tendency of higher values compared with H-FABP (59 %, *p* = 0.064).

### The detail of angiographic findings and invasive treatment

Coronary vessels with significant stenosis (%DS > 75 %) were found at right coronary artery in 25 patients (34 %), left anterior descending artery in 29 (39 %), left circumflex artery in 8 (11 %), and lateral branch in 6 (8 %), and there was no patient with left main trunk disease. Number of the diseased vessels (%DS > 75 %) was one-vessel in 50 (68 %), two-vessels in 13 (18 %), and triple-vessels in 2 (3 %). The type of the culprit lesion for ACS was thrombotic lesion in 34 (46 %). The methods of revascularization were thrombus aspiration for 29, stent implantation for 46, and drug-eluting stent for 8. Final TIMI flow grade was classified to grade 3 in 46 patients, grade 2 in 6, and grade 0 or 1 in none of this study population.

### Diagnostic performance to decide PCI for ACS

To predict angiographic ACS lesions for emergency PCI, the diagnostic performances of the cardiac biomarkers are shown in Table 3. The sensitivity and the NPV of hs-TnT were the highest among the validated biomarkers. The areas under the ROC curves of hs-TnT and H-FABP presented the highest value (0.75); however, these did not reach to the statistical significance in comparison with the other 2 biomarkers. The ROC-optimized cut-off values of hs-TnT, c-TnT, CK-MB, and H-FABP were 18, 11, 3.0, and 4.6 ng/mL, respectively. The rule-in cut-off values were 84, 67, 15.1, and 68 ng/mL, respectively. Although H-FABP showed the highest sensitivity among the validated biomarkers at ROC-optimized cut-off values, the cut-off value of H-FABP was less than the currently used cut-off (6.2 ng/mL). The all rule-in cut-off values of the validated biomarkers showed very low sensitivity. At the ROC-optimized cut-off values, only hs-TnT was appropriate to diagnose angiographic ACS lesions with enough analytical precision.

**Table 3:** Diagnostic performance to predict the angiographic ACS lesion underwent PCI with 95 % CI

% (95% CI)	Cut-off	Sensitivity	Specificity	PPV	NPV	ROC-AUC
hs-TnT						
Current cut-off	14 ng/L	69 % (54–81)^†^	60 % (37–87)	83 % (69–93)	50 % (32–68)	0.75 (0.62–0.88)
ROC-optimized	18 ng/L	63 % (48–76)	74 % (52–90)	84 % (69–94)	47 % (30–65)	
Rule-in cut-off	84 ng/L	12 % (4–24)	100 % (93–100)	100 % (54–100)	53 % (43–63)	
c-TnT						
Current cut-off	30 ng/L	24 % (12–37)^†, ‡^	87 % (77–97)	80 % (52–96)	34 % (22–47)	0.67 (0.54–0.80)
ROC-optimized	11 ng/L	49 % (35–63)	87 % (66–97)	89 % (72–98)	43 % (29–59)	
Rule-in cut-off	67 ng/L	8 % (2–19)	100 % (85–100)	100 % (40–100)	33 % (22–45)	
CK-MB						
Current cut-off	5.0 ng/mL	29 % (17–44)^†, §^	87 % (68–97)	83 % (59–97)	36 % (23–50)	0.68 (0.55–0.81)
ROC-optimized	3.0 ng/mL	63 % (48–76)	70 % (47–87)	82 % (66–92)	46 % (29–63)	
Rule-in cut-off	15.1 ng/mL	2 % (0–10)	100 % (85–100)	100 % (3–100)	32 % (21–43)	
H-FABP						
Current cut-off	6.2 ng/mL	51 % (37–65)^‡, §^	78 % (56–93)	84 % (66–95)	42 % (27–58)	0.75 (0.63–0.88)
ROC-optimized	4.6 ng/mL	71 % (56–83)	78 % (56–93)	88 % (74–96)	55 % (36–72)	
Rule-in cut-off	68 ng/mL	6 % (1–16)	100 % (85–100)	100 % (29–100)	32 % (22–45)	

*ACS* acute coronary syndrome, *PCI* percutaneous coronary intervention, *CI* confidential interval, *PPV* positive predictive value, *NPV* negative predictive value, *ROC* receiver-operator characteristics, *AUC* area under the curve

^†^ *p* value <0.001 in comparison with hs-TnT

^‡^ *p* value <0.01

^§^ *p* value <0.05

### Cardiac markers and the angiographic characteristics

#### Location of the culprit vessel

**Fig. 3:**
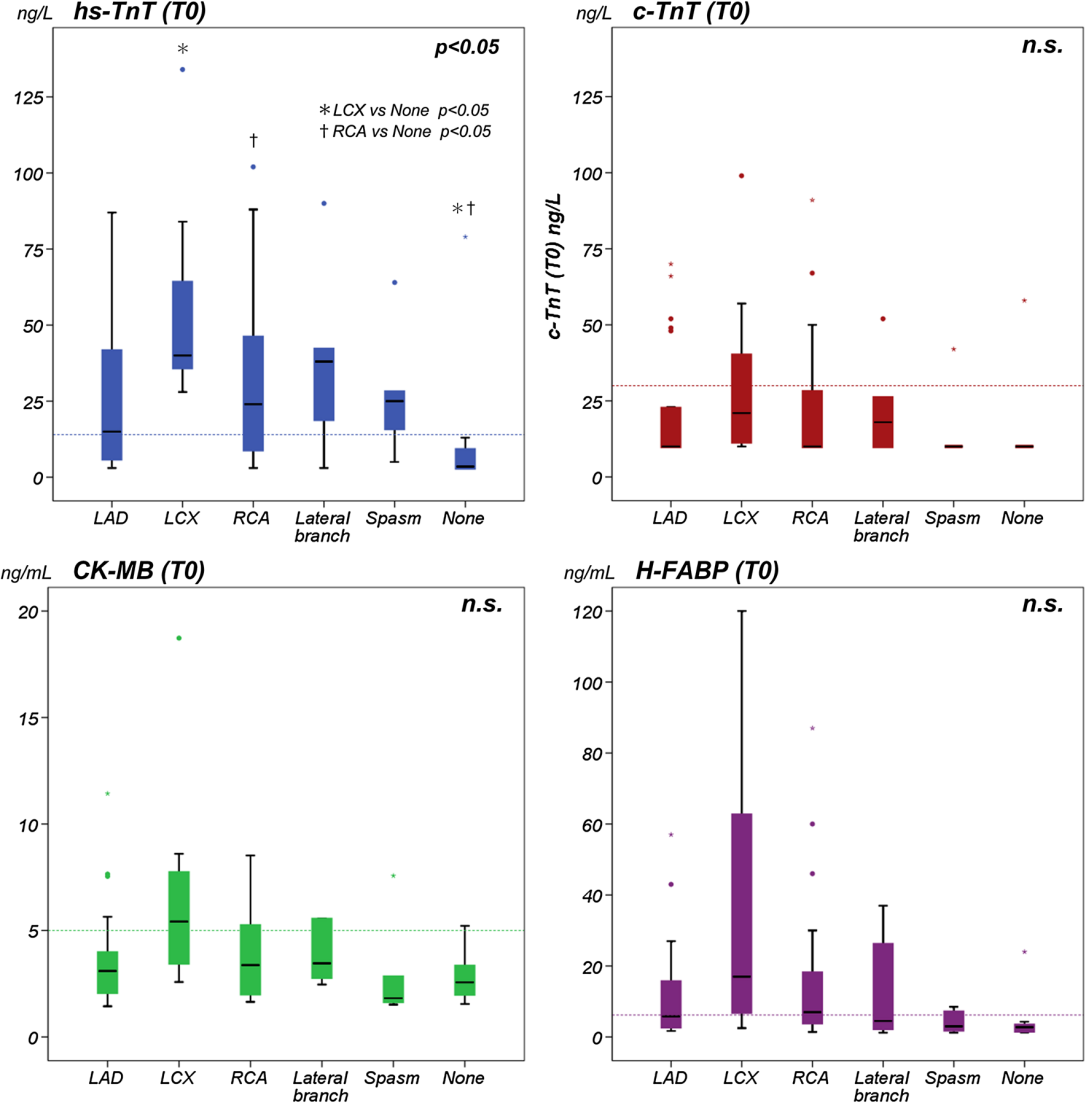
Cardiac biomarkers classified by the location of the culprit vessel

The values of the cardiac biomarkers classified by location of the culprit vessel were presented as box plot (Fig. 3). In multiple comparisons for each cardiac biomarker group, there was statistical significance in the hs-TnT (*p* < 0.05). Patients with left circumflex artery (LCX) and those with right coronary artery (RCA) showed significantly higher value compared with patients without significant stenotic lesion. The patients with LCX admitted later than those with RCA lesion, and there were significant differences in time from the onset to presentation [median (IQR); 250 (173–586) min in LCX vs 165 (100–300) min in RCA, *p* = 0.008].

**Fig. 4:**
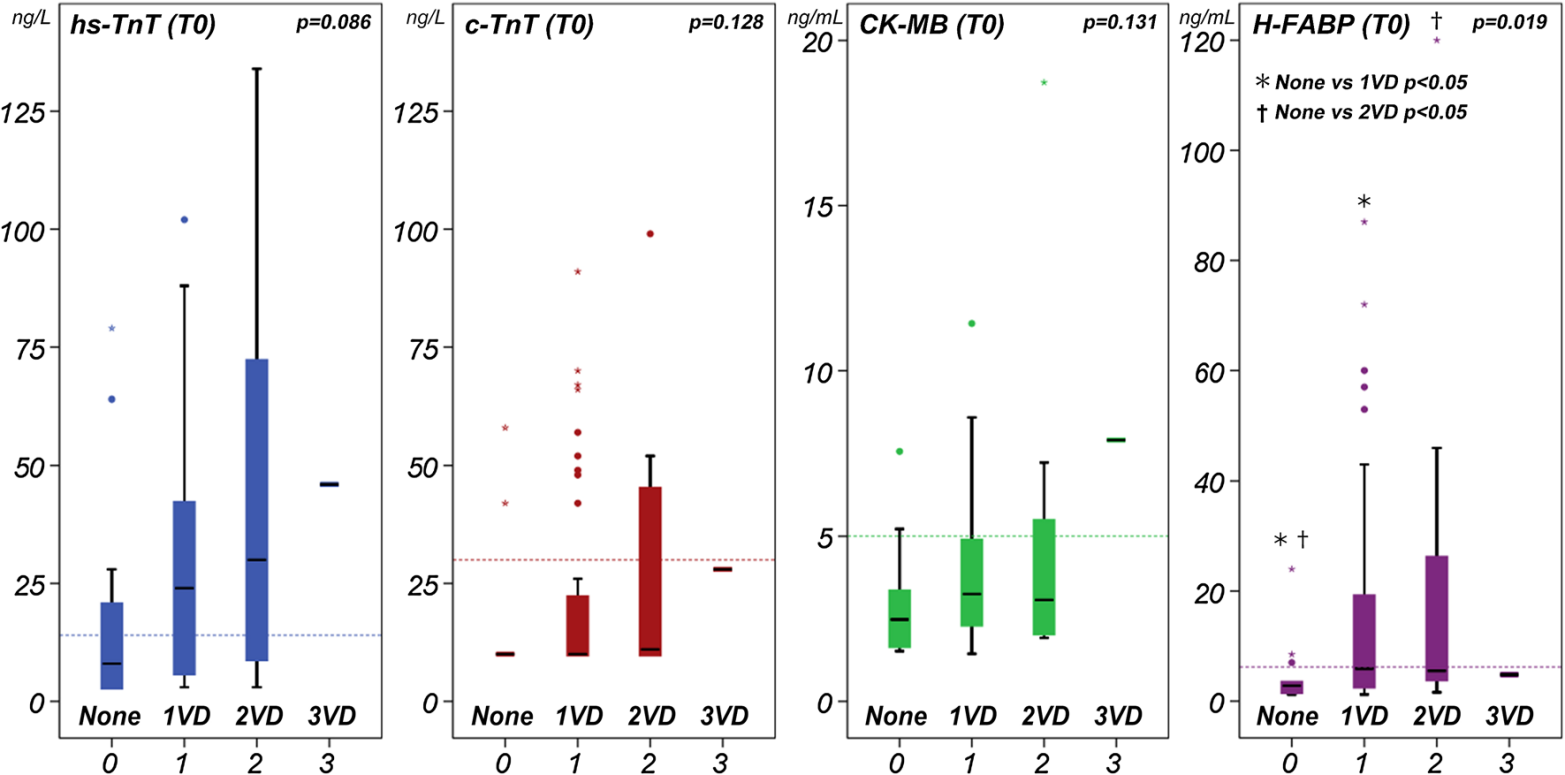
Cardiac biomarkers with stratification by number of the diseased coronary arteries

**Fig. 5:**
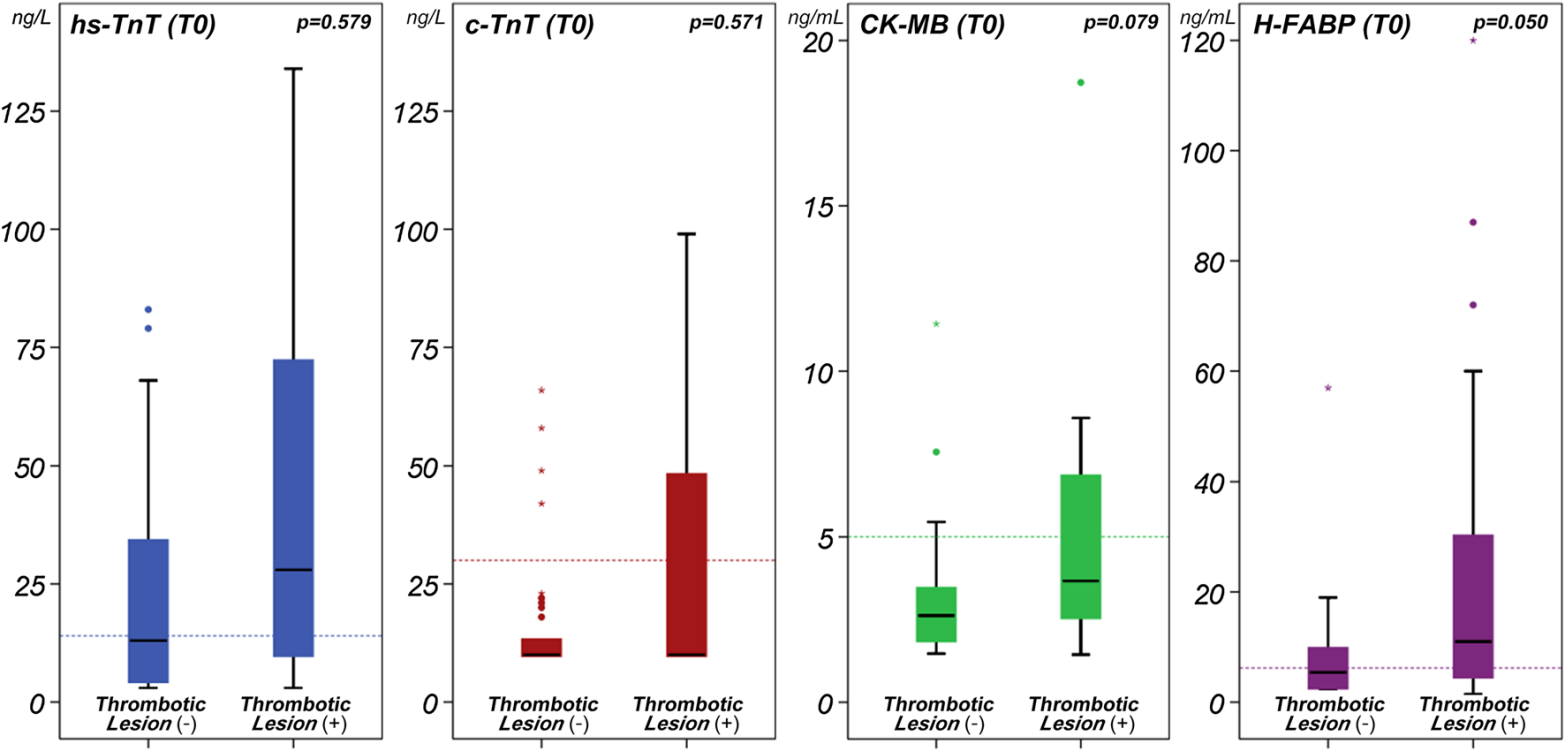
Cardiac biomarkers for the patients having thrombotic lesion

With stratification of the number of the diseased vessels, there was statistical significance only in H-FABP (*p* = 0.019) and a tendency in hs-TnT (*p* = 0.086) (Fig. 4). The values of the cardiac biomarkers were presented as box plot divided by having thrombotic lesion (Fig. 5). Interestingly, H-FABP showed near significantly higher value (*p* = 0.05) in patients with thrombotic lesion compared with those without thrombotic lesion.

## Discussion

### The diagnostic performances to predict angiographic ACS lesion

The present HsTnT-iNET subanalysis has first addressed the correlations between angiographic ACS lesions indicated for emergency PCI and cardiac biomarkers, thereby to clarify the usefulness of the validated biomarkers for the decision making of emergency PCI. Importantly, this study reflected the contemporary clinical practice for ACS, which means that all patients admitted to tertiary cardiovascular center during early phase from onset of chest pain were prospectively enrolled in this study. In this subanalysis, we reviewed the detail of angiographic findings from the raw data of CAG, and investigated the association between the cardiac biomarkers and the characteristics of ACS lesions. Cut-off values of AMI definition had been revised, and recently the 99th percentile value of upper reference limit with coefficient of variance less than 10 % has been recommended in the current guidelines [11–13]. Although the degree of the increase reflected the degree of the risk in the ACS conditions, it seemed uninfluential for the decision of therapeutic strategy to discriminate AMI from the patients with mild increase of cardiac troponin in non ST-segment elevation type ACS. The early invasive strategy has been recommended for the ACS patients with high-risk features such as persistent resting angina pain, TnT >100 ng/L, persistent ST-T deviation, pulmonary edema, and refractory arrhythmia [13].

The present study has provided rational strategy using hs-TnT measurement in the ACS practice. Namely, the present study has demonstrated the newly defined thresholds as the ROC-optimized cut-off value of hs-TnT (18 ng/mL) and the rule-in cut-off value (84 ng/mL). These cut-off values had been established by the most reliable method such as coronary angiography but not cardiac biomarker. Difference in the threshold from 14 to 18 ng/mL may reduce the case number of emergency angiography; meanwhile, the threshold of 84 ng/mL can be used as the absolute indication to achieve early revascularization. Although these thresholds of hs-TnT can be measured with 10 % CV (13.5 ng/mL), the ROC-optimized cut-off values except for hs-TnT were less than the confidential limits to apply. These results suggested that only hs-TnT could be used in such situations.

#### Collateral flow grade and hs-TnT

In the literature, however, there were no reports addressing the relation between the status of collateral flow and the change of cardiac biomarkers. In this study, the values of the cardiac biomarkers were stratified by the collateral flow grades. Patients with total occlusion and poor collateral showed significantly higher values of hs-TnT compared with those in other culprit lesions, which suggested hs-TnT as the most sensitive marker for impending and critical myocardial injury.

#### Elevation of hs-TnT and Myocardial infarction associated with Coronary Spasm

Myocardial infarction caused by coronary spasm has been well known [14–16]. In the investigations by spasm provocation tests for ACS patients [17, 18], more than half of AMI patients were provoked coronary spasm by acetylcholine test, and coronary artery spasm was negligible in the clinical practice of ACS. In the present study, 14 patients were associated with coronary spasm after adjudicating the diagnosis, 4 of 7 patients (57 %) with CAG-proved coronary spasm presented positive hs-TnT, and 2 patients of those were diagnosed as MI. Meanwhile, 4 of 25 patients without significant stenosis (%DS > 75), thrombosis, ulceration, and irregularity were diagnosed as AMI. These patients might be suffered from coronary spasm and should receive the benefits of coronary vasodilator to avoid recurrent ischemia or life-threatening event. The clinical implication of positive hs-TnT in the patients with coronary spasm remained to be established.

#### Differences between hs-TnT and H-FABP for PCI decision

In the present study, both hs-TnT and H-FABP demonstrated powerful diagnostic performance to predict angiographic ACS lesion indicated for emergency PCI. However, there were some differences between hs-TnT and H-FABP. Hs-TnT could detect the patients with severe obstructive stenoses likely to indicate emergency PCI with significantly more accuracy than H-FABP. Second, in ACS patients with  %DS 90–99 % TIMI 3, hs-TnT could diagnose in 91 % of those, with a tendency of higher compared with H-FABP (42 %).

### Limitations

There are several limitations associated with this study. First, all participant institutions were tertiary centers equipped with coronary care units, therefore patients with more severe conditions were likely to be included in this study. In fact, the study cohort had a much higher prevalence of STEMI patients compared with previous reports. Second, the number of patients might be not enough to pursue the significant differences in comparisons for the various findings of CAG. Finally, indication of PCI for the stenotic lesion in the condition with ACS symptom has been largely charged by judgment of the operator, and impact of coronary spasm for ACS could not be determined by CAG. These might be the unavoidable limitation to investigate the correlation between CAG and the cardiac biomarker.

## Conclusions

The present study first demonstrated the correlation between the angiographic culprit lesions for ACS and the cardiac biomarkers. For prediction of ACS lesions requiring emergency PCI, hs-TnT had the highest sensitivity to predict ACS lesions and the newly defined thresholds can provide more strategic implications for angiographic findings. Both hs-TnT and H-FABP discriminated different characteristics of ACS culprit lesions indicated for emergency PCI.

## Abbreviations

ACS: Acute coronary syndrome
AMI: Acute myocardial infarction
CAG: Coronary angiography
CK-MB: Creatine kinase MB isozyme
c-TnT: Conventional troponin T
hs-TnT: High-sensitivity troponin T
HsTnT-iNET: High-sensitivity troponin T for earlier diagnosis of acute myocardial infarction in patients with initially negative troponin T test
H-FABP: Heart-type fatty acid-binding protein
LCX: Left circumflex artery
PCI: Percutaneous coronary intervention
%DS: Percentage diameter stenosis
RCA: Right coronary artery
ROC: Receiver operator characteristics
